# Perceived Detention Environment and Mental Health of Detainees in Immigration Detention Centers in Spain

**DOI:** 10.1007/s40615-024-01977-3

**Published:** 2024-03-22

**Authors:** Virginia Paloma, Isabel Benítez, Armando Agüero-Collins, Carla López-Núñez, Francisco J. Saavedra-Macías

**Affiliations:** 1 Department of Social Psychology, Universidad de Sevilla, Seville, Spain; 2 Department of Methodology of Behavioral Sciences, Universidad de Granada, Spain & Mind, Brain and Behaviour Research Center (CIMCYC), Granada, Spain; 3 The Jesuit Migrant Service, Seville, Spain; 4 Department of Personality, Evaluation and Psychological Treatments, Universidad de Sevilla, Seville, Spain; 5 Department of Experimental Psychology, Universidad de Sevilla, Seville, Spain

**Keywords:** Detention centers, Detention environment, Immigration, Mental health, Spain

## Abstract

The increase in migratory flows worldwide has led to the creation of detention centers as a form of control of irregular migration. Recipient countries are responsible for protecting detainees’ right to mental health, but the literature suggests that immigration detention centers are environments associated with complex mental health needs among the detainees. This study aims to approach the mental health of people detained in the immigration detention centers in Spain, a southern border of Europe. Eighty-seven migrants coming from different Latin American and African countries were interviewed using an adaptation of the Measure of Quality of Life in Detention (MQLD; Bosworth & Gerlach, 2020) to measure the perceived detention environment and The Hopkins Symptom Checklist-25 (HSCL-25; Derogatis et al., 1974) to assess mental health. The results show a high prevalence of detainees with significant levels of anxiety and depression (69%) and attempts at self-harm within the detention centers (19.5%). A more positive perception of the detention environment—especially concerning institutional decency and the relationship with officers—is related to a lower degree of negative mental health symptoms. Finally, people detained for more than 2 weeks assess the detention environment more negatively than those detained for less time. Scientific contributions and social implications to ensure the mental health of detainees from a human rights-based approach are discussed.

## Introduction

During the last decades, there has been an increase in the migratory flow due to issues such as poverty, climate change, armed conflicts, or political problems [1]. This situation—along with a clear political will to increase border controls and maintain inequities between the global North and South—has led to a toughening of migration policies, creating detention centers distributed in various countries worldwide [2]. In the migration context, detention is defined as a “non-punitive administrative measure ordered by an administrative or judicial authority(ies) in order to restrict the liberty of a person through confinement so that another procedure may be implemented” [3]. The purpose of these detention centers is to guarantee the expulsion of an immigrant for being in an irregular administrative situation, for having been previously convicted of a crime, or as a substitute for a criminal conviction [4]. According to the International Organization for Migration [5], an immigrant in an irregular situation refers to a person who is moving or has moved across an international border and is not allowed to enter or remain in a state under the laws of that state and international treaties to which that state is a party. In many countries, detention can also be used against asylum-seekers, who can be detained upon arrival for purposes other than expulsion, such as detention pending identity verification. Immigration detention centers have been considered a powerful tool of structural violence and control by receiving countries in the North against people suffering from global inequities [6, 7].

Despite the “non-punitive” character contemplated in the receiving countries’ official narratives, emerging fields such as border criminology or carceral geography conceive immigration detention centers as a form of deterrence, with bureaucratic processes and architectural features associated with punishment, arguing that the confinement is experienced by the detainees as deliberately punitive [7–11]. In fact, the literature shows that immigration detention centers are environments associated with complex mental health needs among detainees [12]. Immigrants who enter detention centers face—in an unplanned way—the dynamics characterized by loss of agency and liberty, depersonalization, loneliness, confusion, uncertainty about their situation, communication difficulties with the immigration staff and lawyers, and feelings of humiliation concerning other detainees or staff [6]. As Cleveland and Rousseau [13] state, “detainees have little to do except think about their problems, worry about being deported, and about the well-being of their family back home” (p. 414–415). Faced with this situation, the state is responsible for guaranteeing detainees their right to the highest possible level of mental health. In this sense, the United Nations [14] claims that “States have a tripartite obligation to respect, protect, and fulfill the right to mental health, […] particularly for those in the most disadvantaged situations” (Article 18). Therefore, it is necessary to properly understand the detainees’ psychological experience in detention centers to address their needs and guarantee their right to mental health within these closed settings.

This study aims to approach the mental health of detainees in immigration detention centers in Spain. To our knowledge, there is no study carried out in Spain on this subject, despite Spain’s being a strategic geographical enclave of reference in managing immigration on the southern border of Europe. Next, the mode of operation of the immigration detention centers in Spain is contextualized, and the international literature linking immigrant detention with mental health is reviewed. Then, the objectives of this study are defined, and the method used to achieve them is described. Finally, the results obtained are shown, and the scientific contributions and social implications to ensure detainees’ mental health from a human rights-based approach are discussed.

### Immigration Detention Centers in Spain

Detention centers in Spain are called “Centers of Internment for Foreigners” and were created in 1985. They are supervised by the Ministry of the Internal Affairs of the Spanish government, managed by the General Police Directorate, and some services are provided by the private sector, such as medical services [4]. There are currently seven detention centers located in Madrid, Valencia, Barcelona, Las Palmas, Algeciras, Murcia, and Tenerife [15]. In 2022, in Spain, there were 2276 inmates (1.9% women), of whom 53.1% were finally expelled [16]. The maximum number of days that a person can remain in a detention center in Spain is 60. This national regulation is more guarantee-based than the European one, which establishes the maximum period at 6 months, but it can be extended to 18 months in some cases [17]. In any case, the average length of detention in Spain is between 20 and 30 days [18], with an average of 30.2 days in 2022 [16]. According to the law, minors can be kept in detention centers as part of the family unit, but this possibility is not being used in practice [18]. Unaccompanied minors cannot be detained. Instead, they are placed in public facilities for the protection of minors. Finally, asylum-seekers cannot be detained in Spain. However, immigrants who have applied for international protection during their detention remain in detention centers until the outcome is known [4].

Despite various amendments made to the law to improve the living conditions of the inmates, the Ombudsman of Spain and several social organizations have denounced the deficient conditions in detention centers, violations of the inmates’ rights, and the low quality of life within them [4, 16]. For example, it is worth highlighting how the national police manage the centers, which grants them a prison-type functioning, together with (a) insufficient educational, social, and leisure areas and activities; (b) deteriorated infrastructures; (c) emotional fatigue of some staff members; (d) the absence of interpreters to ensure good communication between workers and inmates; or (e) the absence of notification on the progress of the file, generating high levels of uncertainty [15, 17, 19, 20]. In addition, these centers have a deficit of health care through an outsourced service that does not include psychological care service in its specifications, despite the inmates’ psychological suffering observed by various social actors [16, 17, 21].

### Immigration Detention Centers and Mental Health

The international literature suggests that—in addition to exacerbating the detainees’ former problems—detention centers have a significant negative impact on the inmates’ mental health and should be considered a traumatic experience in itself [12, 22–27]. The systematic review performed by Werthern et al. [27], including twenty-six relevant studies conducted in Australia, Canada, Israel, Japan, Sweden, Switzerland, the UK, and the USA, showed that anxiety, depression, and post-traumatic stress disorder were the most commonly reported disorders during and after detention. Indeed, there is evidence of how detention causes long-term psychological damage, often with persistent negative symptoms such as a lingering sense of insecurity, difficulties in relationships, persistent depression and anxiety, or intrusive memories for a long time after the detention [26, 28]. Esposito et al. [22], working in the context of detention centers in Italy, also drew attention to the processes of autolysis and suicidal ideations observed among the inmates. Specifically, Hedrick et al. [29] found that rates of self-harm among asylum-seekers in immigration detention centers were four times higher than rates among asylum-seekers outside detention centers and 22 times higher than in the general population in Australia. Indeed, a recent systematic review focused on asylum-seekers, and refugees in Europe supports the idea that detention centers are particularly “risky” contexts for self-harm practices [30]. This deterioration in mental health has been observed by the detainees themselves, as well as by health professionals who have attended to immigrants in immigration detention centers across the USA. From their perspective, “the detention experience itself was linked with worsened psychological symptoms” [23, p. 6].

Authors like Van Hout et al. [31] found that conditions in immigration detention centers are often described as “inhumane, resembling prison, and underpinned by communication difficulties, lack of adequate nutrition and responsive health care” (p. 221). For this reason, different studies have analyzed the impact of the detention environment on detainees’ mental health [32]. Saadi et al. [33] found that a higher number of confinement conditions experienced in detention centers (measured by sleep deprivation, difficulty accessing family visits, harassment, witnessed harassment, difficulty accessing medical services or psychological services) were associated with poorer mental health among detained immigrants in California (USA). This study highlights the cumulative impact and the important role of the environment on detainees’ mental health. Along the same line, Puthoopparambil et al. [34] found that the low support received from staff in the centers was the main factor explaining the low perceived quality of life among immigrants in Swedish detention centers.

Finally, different studies have found a positive relationship between the severity of negative mental health symptoms and the length of stay in detention centers [24, 27, 35–37]. For example, Steel et al. [26] found that refugees who had been confined for 6 months or more had more severe mental health symptoms than those who had been detained for less time in Australia (although they also reported significant stress). However, other studies did not find this linear relationship between detention time and mental health [38, 39]. The literature indicates that immediately upon release, detainees experience an initial improvement in mental health through improved psychological functioning, reduced suicidal ideation, and a higher perceived quality of life [27, 40].

Based on the above, the objectives of this study are (a) to evaluate the level and the most common symptoms of mental health (i.e., anxiety, depression, and self-harm) of detainees in three immigration detention centers in Spain, to analyze this measure according to sociodemographic variables, and to explore the participants’ disposition to use psychological services if they were available in the centers; (b) to analyze how the detainees perceive the detention environment (in terms of institutional decency, health care, security, immigration staff and lawyers, detainee cohesion, and relationship with officers) and how it is related to their mental health; and (c) to analyze differences in mental health and perceived detention environment according to time spent in detention.

## Methods

### Participants

The recruitment process of the participants was carried out through a convenience selection from the list of inmates that the managers of the detention center offered at the beginning of each visit, guaranteeing access to diverse profiles in terms of country of origin, age, and length of stay in the center. Specifically, the selected person had to meet the following inclusion criteria: (a) being detained at the time of the evaluation in the detention center in Madrid, Valencia, or Algeciras; (b) could understand and express themselves in Spanish or in the language of the person acting as interpreter (i.e., Arabic), if any; and (c) voluntarily expressed a desire to participate in the study.

The sample comprised 87 participants, 83 men and 4 women (see Table 1). The mean age was 30.92 years (age range 19–52 years, SD = 8.55). The level of education attained was as follows: none (21.8%), primary education (33.3%), secondary education (40.2%), and higher education (3.4%). Most of the participants were single (71.3%) and came from Morocco (57.5%), Colombia (13.6%), and other Latin American and African countries (28.9%). The length of stay in Spain ranged from less than 1 year to 23 years (*M* = 5, SD = 5.51), whereas the days of stay in the detention center ranged between 1 and 55 days (*M* = 19.38, SD = 14; it should be noted that the maximum detention period in Spain is 60 days). Most participants were detained because of an irregular stay in the country (57.5%), an unauthorized arrival (18.4%), or after having lost their right to remain in Spain because they had been convicted of committing a crime (23%). As a result, all participants were undocumented. We assessed the participants in three detention centers: Algeciras (47.1%), Valencia (27.6%), and Madrid (25.3%).

**Table 1:** Sociodemographic characteristics of the participants

		*n*	Percentage (%)
Gender	Male	83	95.4
	Female	4	4.6
Educational level	No studies	19	21.8
	Primary studies	29	33.3
	Secondary studies	35	40.2
	Higher education	3	3.4
	Missing values	1	1.1
Civil status	Single	62	71.3
	With partner	20	23
	Separated	2	2.3
	Missing values	3	3.4
Country of origin	Argentina	6	6.9
	Brazil	2	2.2
	Chile	1	1.1
	Colombia	12	13.6
	Cuba	1	1.1
	Ecuador	3	3.4
	Georgia	1	1.1
	Honduras	1	1.1
	Marroco	51	57.5
	Paraguay	1	1.1
	Perú	4	4.6
	Czech Republic	1	1.1
	Dominican Republic	3	3.3
	Senegal	1	1.1
Reason for Internment	Unauthorized arrival	16	18.4
	Irregular stay	50	57.5
	Crime	20	23
	Missing values	1	1.1

### Instruments

Participants responded to several sets of questions contained in an assessment protocol. Specifically, the protocol consisted of three sections.

Sociodemographic data. This section asks about the categories already described in the “Participants” section.

Perceived detention environment. We measured this variable through an adaptation of the Measure of Quality of Life in Detention (MQLD) [41], an instrument specifically developed by the University of Oxford to measure the quality of life perceived by immigrants within detention centers. Specifically, the version adapted for this study has 18 items that measure the detainee’s perception of six dimensions: (a) institutional decency (e.g., “*This center is clean*”), (b) health care (e.g., “*Healthcare staff believe me*”), (c) security (e.g., “*I feel safe in my room*”), (d) immigration staff and lawyers (e.g., “*I can call my lawyer when I need to*”), (e) detainee cohesion (e.g., “*I trust most of the other detainees here*”), and (f) relationship with officers (e.g., “*Officers and detainees get along well here*”). The answers are rated on a four-point Likert scale (*never, sometimes, most of the time, always*). The final score of each item can range from 1 to 4, in each dimension from 3 to 12, and the total scale score ranges from 18 to 72, with a higher value reflecting a better rating (2.5, 7.5, and 45 are the neutral cutoff points as median scores, respectively). The Cronbach alpha value for this study was 0.89.

Mental health. To assess the anxious and depressive symptoms presented by the detainee, we used The Hopkins Symptom Checklist-25 (HSCL-25) [42]. This instrument evaluates the presence of anxiety and depression through two subscales, consisting of 10 items (e.g., “*feeling fearful*”) and 15 (e.g., “*feeling blue*”), respectively. The Cronbach alpha values for this study were 0.89 for anxiety and 0.88 for depression. The response scale is a four-point Likert type (*not at all, a little, quite a bit, extremely*). The final score of each item, each subscale, and the total scale can range from 1 to 4, with a value closer to 4 reflecting worse mental health. This instrument has been widely used with immigrants worldwide, using a cutoff value of 1.75 for a total score based on the average of both depression and anxiety. Participants scoring higher than 1.75 are defined as “a clinically distressed case” [13, 26, 28, 43–45]. In this study, we used the adaptation to Spanish by Clavería et al. [46]. In addition, we asked the detainee when these symptoms had begun (before or after entering the detention center). To ascertain the existence of self-harm, we asked the detainees whether they had attempted to harm themselves during their stay in the detention center (yes or no). The protocol also included one final open question asking whether participants would use psychological services if available.

### Procedure

For this study, our research group established a collaboration agreement with the Jesuit Migrant Service, one of the leading social organizations in Spain that regularly visits detainees to monitor and denounce their situation in immigration detention centers. This community partner has been key to our research team in developing a research agenda that responds to community needs, gaining access to participants, and making our findings visible to stakeholders and society at large.

For reasons of linguistic difficulty and/or low educational level of some of the participants, this study chose oral administration with a semi-structured questionnaire. The evaluations took place from September to December 2022 and were conducted by the first author together with previously trained professionals and with the help of an interpreter when necessary. This data collection process was not affected by any exceptional measures related to the COVID-19 pandemic. The administration lasted between 30 and 45 min and was conducted in a private interview room in the visitors’ area of the detention center. Concerning the language, 56.8% of the evaluations were conducted in Spanish, 40.9% in Arabic, and 2.3% in Arabic-Spanish. Before the session, we explained to the detainee the voluntary nature of participation in the study, their right to stop the assessment whenever they wanted, and other relevant ethical issues. This information was specified in an informed consent document that the person accepted before starting the session.

Descriptive analyses were conducted to identify the most common symptoms and the perceptions of the most limited dimensions in the detention environment. On the HSCL-25, the mean was used as the total score, but also to identify participants above the clinical cutoff (those with mean scores greater than 1.75). Sociodemographic variables were used to analyze differences in participants’ total scores. The MQLD provided a total global score as well as total scores for each dimension. We computed correlations to understand the relationships between the dimensions and the symptoms assessed by the HSCL-25, after checking the linearity assumption. Finally, we conducted an ANOVA to evaluate potential differences in mental health and the perceived detention environment among participants with different lengths of stay.

## Results

### Mental Health of Detainees

First, the analysis focused on evaluating the level and most common symptoms of mental health expressed by detainees. Results indicate that 69% of the sample scores were above the cutoff value of HSCL-25 (see Table 2). In 71.3% of cases, these symptoms began during the stay in the detention center. Among the most frequent symptoms in the anxious dimension were “nervousness” (*M* = 2.7, SD = 1.06), “feeling restless” (*M* = 2.69, SD = 1.17), and “feeling tense” (*M* = 2.58, SD = 1.15), while in the dimension of depression, they were “feeling trapped” (*M* = 3.12, SD = 1.12), “feeling lonely” (*M* = 2.92, SD = 1.13), “worrying too much” (*M* = 2.91, SD = 1.12), “feeling blue” (*M* = 2.60, SD = 1.17), and “sleep disturbance” (*M* = 2.58, SD = 1.34), within a possible range of 1 to 4 (see Table 2).

**Table 2:** Mental health indicators among participants in the study

Mental health	Mean (1–4)
HSCL-Anxiety	**2.26** (SD = 0.8)
1. Being scared for no reason	1.72
2. Feeling fearful	2.32
3. Faintness	2.23
4. Nervousness	2.7
5. Heart racing	1.84
6. Trembling	1.68
7. Feeling tense	2.58
8. Headache	2.16
9. Feeling panic	1.92
10. Feeling restless	2.69
HSCL-Depression	**2.32** (SD = 0.7)
11. Feeling low in energy	2.37
12. Blaming oneself	2.38
13. Crying easily	1.93
14. Losing sexual interest	1.74
15. Feeling lonely	2.92
16. Feeling hopeless	2.14
17. Feeling blue	2.60
18. Thinking of ending one’s life	1.34
19. Feeling trapped	3.12
20. Worrying too much	2.91
21. Feeling no interest	2.18
22. Feeling that everything is an effort	2.38
23. Worthless feeling	1.92
24. Poor appetite	2.01
25. Sleep disturbance	2.58
Total HSCL-25	**2.31** (SD = 0.69)
Diagnosis	**Percentage (%)**
Not a case	25.3
Above the clinical cutoff	69
Missing data	5.7
Onset of symptoms	**Percentage (%)**
Before entering the detention center	13.8
During the stay	71.3
Missing data	14.8
Attempting self-harm inside the detention center	**Percentage (%)**
No	69
Yes	19.5
Missing data	11.5

*HSCL-25* The Hopkins Symptom Checklist-25 (Derogatis et al., 1974)

Moreover, 19.5% of the sample reported having tried to harm themselves during their stay in the center (see Table 2). When we compare the profile of detainees who report having tried to harm themselves with those who do not, we find that the former manifest a greater degree of anxiety and depression, as well as a lower sense of security and a poorer relationship with officers within the immigration detention center (see Table 3).

**Table 3:** Differences between participants who have tried to harm themselves and those who have not

	Non-self-harm participants	Self-harm participants	*t*
HSCL-Anxiety	2.16	2.56	− 1.86*
HSCL-Depression	2.23	2.72	− 2.70**
Total HSCL-25	2.21	2.66	− 2.46**
MQLD-Institutional decency	8.39	7.76	0.77
MQLD-Health care	9.59	8.53	1.56
MQLD-Security	10.23	8.65	2.41**
MQLD-Immigration staff and lawyers	7.49	7.18	0.38
MQLD-Detainee cohesion	8.4	8.12	0.38
MQLD-Relationship with officers	9.32	7.59	2.57**
Total MQLD	52.13	47.82	1.57

*HSCL-25* The Hopkins Symptom Checklist-25 (Derogatis et al., 1974), *MQLD* Measure of Quality of Life in Detention (adaptation from Bosworth & Gerlach, 2020)

^*^p < .05; **p < .01

We found that the mental health—measured through the HSCL-25—does not differ in terms of age (19–35, 36–52, *t* =  − 0.355, *p* = 0.36), length of stay in Spain (0–2, 3–10, 11–23, *t* = 2.41, *p* = 0.10), region of origin (Latin America, Morocco, other, *F* = 0.152, *p* = 0.86), educational level (*F* = 0.564, *p* = 0.64), civil status (*F* = 2.965, *p* = 0.06), detention center (Algeciras, Madrid, Valencia; *F* = 0.391, *p* = 0.76), or reason for detention (*F* = 1.37, *p* = 0.25).

Regarding the psychological services, 87% of the participants declared they would use them if they were available in immigration detention centers, supporting the idea that these services meet a need and would be well received by detainees. Specifically, participants formulated statements such as the following: “*Psychological services would be a relevant support to work with my feelings*;” “*I think it is a totally needed tool*;” “*Some people here feel very bad, and they need help*;” or “*I think it would be positive to improve my situation.*”

### Perceived Detention Environment and Mental Health

When analyzing how the detention environment is perceived by detainees, we found that the total average score on the MQLD was 50.34, with a possible range of 18 to 72 and a median score of 45. Complementarily, when examining each of the dimensions of the MQLD, we found that most people scored above the average (7.5, within a possible range of 3 to 12; see Table 4). This means that their perception of the detention environment was generally slightly above the neutral value of the median score. The highest rated dimension was “security” (*M* = 9.83, SD = 2.46), referring to how detainees generally felt safe within the detention center facilities. The worst-rated dimension was “immigration staff and lawyer,” referring to the poor access and communication with lawyers in a language they could understand and being poorly informed of changes in their file (*M* = 7.44, SD = 2.96; see Table 4).

**Table 4:** Perceived detention environment among participants in the study

	Mean
MQLD-institutional decency	**8.15** (SD = 2.94)
This center is clean	3.02
I have enough clothes	2.93
The food here is good	2.5
MQLD-Health care	**9.23** (SD = 2.96)
Healthcare staff believe me	3.08
I can see a doctor when I need to	3.39
Healthcare staff treat me with respect	3.28
MQLD-Security	**9.83** (SD = 2.46)
I feel safe around other detainees here	3.27
I feel safe in my room	3.41
I feel safe in the dining hall and other common areas	3.3
MQLD-Immigration staff and lawyers	**7.44** (SD = 2.96)
I can call my lawyer when I need to	3.01
I know what is happening in my immigration case	2.26
Staff here can help explain my case in a language I understand	2.89
MQLD-Detainee cohesion	**8.13** (SD = 2.76)
I trust most of the other detainees here	2.83
I like to spend time with other detainees	2.75
Detainees from different countries get along well here	2.85
MQLD-Relationship with officers	**8.81** (SD = 2.87)
I can talk to officers when I need to	3.08
Most officers in this center are kind to me	3.13
Officers and detainees get along well here	2.89
Total MQLD	**50.34** (SD = 13.30)

*MQLD* Measure of Quality of Life in Detention (adaptation from Bosworth & Gerlach, 2020)

On the other hand, there was a correlation of − 0.30 between the MQLD total score and the HSCL-25 total score (*p* < 0.01; see Table 5). This means that a more positive perception of the detention environment is related to lower negative mental health symptomatology (anxiety and depression). Within the perceived detention environment, two dimensions stand out as especially relevant for their relationship with mental health: institutional decency (*r* =  − 0.32, *p* < 0.01) and the relationship with officers (*r* =  − 0.27, *p* < 0.05). Thus, better institutional decency (e.g., perceiving that the center is clean or that the food is good) and better relations with the officers (e.g., perceiving a good relationship between police officers and detainees or that the officers are kind to the detainees) are linked to fewer negative mental health symptoms.

**Table 5:** Correlations between mental health, perceived detention environment, and time spent in the detention center

Variables	(1)	(2)	(3)	(4)	(5)	(6)	(7)	(8)	(9)	(10)	(11)
Length of stay (1)		N/A	N/A	N/A	− .2	− .28*	− .29**	N/A	N/A	− .27*	− .33**
HSCL-Anxiety (2)			.72**	.92**	− .29**	− .2	− .15	− .17	− .05	− .28*	− .28*
HSCL-Depression (3)				.94**	− .31**	− .24*	− .2	− .12	− .11	− .27*	− .29**
Total HSCL-25 (4)					− .32**	− .19	− .2	− .17	− .08	− .27*	− .3**
MQLD-Institutional decency (5)						.48**	.4**	.33**	.32**	.55**	.66**
MQLD-Health care (6)							.45**	.23*	.45**	.53**	.77
MQLD-Security (7)								.24*	.59**	.46**	.71**
MQLD-Immigration staff and lawyers (8)									.18	.22*	.55**
MQLD-Detainee cohesion (9)										.47**	.7**
MQLD-Relationship with officers (10)											.7**
Total MQLD (11)											

N/A: Correlation analyses were not conducted in this situation as the linearity assumption was not satisfied

*HSCL-25* The Hopkins Symptom Checklist-25 (Derogatis et al., 1974), *MQLD* Measure of Quality of Life in Detention (adaptation from Bosworth & Gerlach, 2020)

^*^p < .05. **p < .01

### Length of Stay, Perceived Detention Environment, and Mental Health

Significant correlations appeared when assessing the relationship between time spent in the center and perceived detention environment (*r* =  − 0.33, *p* < 0.01; see Table 5). In particular, there were two dimensions of the detention environment in which participants with different lengths of stay differed: health care and relationships with officers (see Table 6). Thus, participants who stayed longer than 7 days assessed the officers and health professionals more negatively than those who stayed 7 days or less. This reduced the scores on the total MQLD scale for participants who stayed longer than 2 weeks, indicating the lowest quality of life for these participants in the detention center.

**Table 6:** Mental health and perceived detention environment depending on the time of stay

Variables	Mean 0–7 days group (*n* = 22)	Mean 7–14 days group (*n* = 22)	Mean 15–30 days group (*n* = 23)	Mean 31 or more days group (*n* = 20)	*F*
HSCL-Anxiety					.36
HSCL-Depression					1.03
Total HSCL-25					.41
MQLD-Institutional decency					2.32
MQLD-Health care	11.36^**1 > 3, *1 > 2, 1 > 4^	8.82	8.04	8.63	6.42**
MQLD-Security					2.4
MQLD-Immigration staff and lawyers					.93
MQLD-Detainee cohesion					.92
MQLD-Relationship with officers	10.45^*1 > 3^	8.41	8.04	8.32	3.55*
Total MQLD	58.73	50.68^**2 > 3; *2 > 4^	46.35	47	4.85**

*HSCL-25* The Hopkins Symptom Checklist-25 (Derogatis et al., 1974), *MQLD* Measure of Quality of Life in Detention (adaptation from Bosworth & Gerlach, 2020)

^*^p < .05; **p < .01

On the contrary, correlations between length of stay and mental health (anxiety, depression, and total score) were not calculated, as linearity tests indicated that the relationship between them was nonlinear (see Table 5). In a complementary approach, we compared the groups divided by length of stay. However, no significant group differences were found (see Table 6).

## Discussion

This study takes a novel stance on the mental health of detainees in immigration detention centers in Spain. Firstly, this study found many detainees with anxiety and depression levels above the clinical cutoff, indicating that they are experiencing high levels of distress that warrant further investigation, that is, a full clinical assessment to determine whether they should be diagnosed with a disorder requiring treatment. In addition, 19.5% of the participants said they had tried to harm themselves during their stay in the center. These results are consistent with the international literature implying that detention centers have a negative impact on the inmates’ mental health [12, 22, 24–26] and supporting the literature that suggests that detention centers are risky scenarios for self-injurious practices [30]. We highlight the relevance of our results because they expand the evidence that this negative impact on mental health occurs among immigrants—independently of age, length of stay in Spain, region of origin, educational level, civil status, location of the detention center, or reason for detention—and during relatively short periods of detention (19 days on average in our case). It should be noted that most studies conducted on mental health in detention centers are based on a particularly vulnerable profile (i.e., asylum-seekers) of people who are detained for long periods of time, usually months or even years [28, 40]. In this way, our results reinforce and expand the idea that detention centers are, in themselves, a threat to the detainees’ mental health. They also emphasize the need to provide psychological support, a service highly requested by detainees.

Secondly, this study has found that factors relating to the detention environment can adversely affect mental health. Specifically, we found that a more positive perception of the detention environment—especially regarding institutional decency (cleanliness, food) and the relationship of respect with officers—is related to a lower degree of negative symptoms of anxiety and depression. These results empirically support the recent and still emerging literature on the key role of detention conditions for detainees’ mental health [32, 33] and specify two key elements to be considered by public health policies, as discussed below in the social implications section. In this way, poor mental health can be regarded as a predictable response to a scenario where detainees often experience multiple intersecting vulnerabilities [47].

Thirdly, we found that the perception of the detention environment is worse among those detained for longer periods. In particular, people who have been confined for more than 7 days make a worse assessment of the health service personnel and police officers compared to those who have been detained for less than 7 days. As a result, after 2 weeks of detention, the overall perception of conditions in the detention center worsens significantly. To our knowledge, this is a novel result in the scientific literature, possibly due to different reasons. In particular, detention centers often evoke detainees’ loneliness and feelings of abandonment [48], and a tendency to perceive a lack of care from others [49]. In addition, waiting in this detention environment could be conceptualized as an exercise of power, where detainees are subordinated to the will of others [50]. In this context, this 2-week rupture can be linked to detainees’ hopelessness and despair in the face of a situation of perceived lack of protection from officers and health professionals, which inevitably increases as the days in the detention center go by.

On the contrary, the results of our study do not support a significant relationship between the length of detention and mental health, a result found in other studies [24, 36, 37]. This may be because the maximum detention time allowed in Spain is 60 days, and this may be too short to identify significant changes in mental health. For example, Essex et al. [35] established 3 months as the cutoff point for markedly worsening mental health. Moreover, precedent research has shown that the impact of detention on mental health is exacerbated when detainees do not know in advance the maximum length of stay in the detention center, a situation that does not exist in Spain. Uncertainty is well known to be one of the most significant sources of stress [51]. For example, immigration detention can be indefinite in countries such as the UK [50, 52, 53], Australia [48], or Canada [6], resulting in a profound state of uncertainty that inevitably leads to hopelessness and powerlessness [6, 52]. In particular, the hopelessness associated with this indefinite stay increases over time [48], aggravating detainees’ sense of loss of control over their own lives and complete uncertainty about their future [6, 52, 53]. Unlike prisoners or detainees who know in advance their release date (i.e., as in Spain), individuals in this situation of temporary uncertainty feel that an unexpected change could occur every day and that, ultimately, they are stuck in the system [53]. This endless wait and unpredictability about their release, added to the fact of not knowing how their detention will end [50], are factors associated with a worsening of the detainees’ mental health [6]. Considering these findings, having a known maximum waiting time in Spanish detention centers may have partially preserved detainees’ mental health over time. In any case, longitudinal studies are needed to accurately assess the impact of the passage of time on mental health.

### Limitations and Future Research

This study has some limitations. First, the time established for visits in these centers is quite limited, which makes it challenging to carry out a more detailed analysis of the mental health of each detainee or to make use of other complementary assessment instruments. For example, although our study made a first approach to self-harm practices, we agree with Gargiulo et al. [30] when establishing that “the complex meanings that self-harm practices might have in detention (e.g., from help-seeking to political resistance) need to be further investigated” (p. 195). Likewise, the data collected are based solely on the detainees’ perceptions. Secondly, we have not explored relevant variables such as substance use, although other studies have found a high prevalence in detention centers [54], or perceived social support, despite that other studies found more mental health problems among more isolated detainees [27]. Moreover, more research is needed on the detention environment and its relationship with psychological variables. The concept of “torturing environments” and measures such as the “Torturing Environment Scale” could be useful in this endeavor [11, 55]. In general, there is a need for more qualitative and intersectional approaches to the study of immigration-related detention environments, including variables such as language barriers or experiences of racism. Thirdly, although access to detained immigrants is challenging to achieve, our sample may be considered small, especially in the case of women. Although this low proportion in our sample reflects the gender distribution existing within detention centers in Spain, this has precluded exploring our results according to gender. However, this is important for future research because “the mental health consequences of detention for female detainees specifically […] remain unknown based on the studies reviewed [after carrying out a systematic review]” [27, p. 12].

### Social Implications

The results of this study suggest the need to (a) introduce a psychological accompaniment service within detention centers, which currently does not exist in the Spanish context; (b) improve the conditions of the detention environment, especially concerning the conditions of the stay, the relationship with officers, and communication with lawyers; and (c) reduce waiting times within detention centers or choose alternative measures to detention.

Concerning the first point, we agree with Van Hout et al. [31] on the need to “strengthen the provision of culturally sensitive health services and competent health workers, specialized in migrant health, within immigration detention settings” (p. 232). These mental health care services in detention centers must be provided from a human rights-based approach, ensuring that the human rights of immigrants receiving services are protected, promoted, and supported by staff [56]. The FREDA principles focus specifically on five human rights that need to be promoted: (a) fairness (e.g., detainees have the right to receive information in a format they can understand), (b) respect (e.g., detainees have the right to feel valued by the staff), (c) equality (e.g., detainees have the right not to be discriminated against because of their characteristics), (d) dignity (e.g., detainees have the right to be treated as human beings), and (e) autonomy (e.g., detainees have the right to live according to personal values, beliefs, and preferences) [56, 57].

Concerning the second point, this study indicates the importance of improving the conditions of the stay (cleanliness, clothes, food, among others) and the relationship with the officers due to their link with the detainees’ manifested mental health. As several authors argue [58], the work inside detention centers is challenging and emotionally demanding, suggesting the appropriateness of promoting ongoing group spaces of support, dialogue, and self-reflection among the detention staff and officers. This is important, considering that the officers’ welfare—as an integral part of the detention environment—and the detainees’ welfare are interdependent [59]. At this point, it is also necessary to highlight the need to improve communication between detainees and lawyers, as it was the worst-valued dimension within the detention environment. This is in line with the findings of Puthoopparambil et al. [60] in Swedish detention centers, where detainees reported not receiving adequate information and, as a result, experiencing a lack of control over their own lives.

In relation to the third point, given the negative impact that the length of stay has on the perception of quality of life within detention centers, this study suggests the need to advocate for minimizing the waiting times. In any case, we agree with Zion et al. [61] that “without systemic change, there is no way to address the mental suffering caused by the detention setting” (p. 74). In this sense, we advocate for the majority use of alternatives to detention in the long term, defined as “non-custodial measures used to monitor and/or limit the movement of third-country nationals in order to ensure compliance with international protection and return procedures” [3]. In Spain, the alternatives to detention include regular reporting (i.e., reporting to the police at regular intervals), staying in a specific place, and surrendering travel or identity documents (which are kept at the police station). Moreover, there is an important precedent for the abolition of detention when—due to the COVID-19 pandemic—the Spanish government released all detainees and closed all immigration detention centers in its territory for almost five months in 2020 [62].

We hope that the results obtained in this study will visibilize the reality of mental health within detention centers, advocate for the detainees’ mental health as a human right, and guide the development of policies and practices that are not detrimental to immigrants’ mental health.

## Data Availability

The data that support the findings of this study are available on request from the corresponding author.
